# Azoospermia factor microdeletions: common tag STSs in infertile men with azoospermia and sever oligospermia from Egypt

**DOI:** 10.1186/1471-2164-15-S2-P25

**Published:** 2014-04-02

**Authors:** Nasser A Elhawary, Neveen S Seif-Eldin, Marwa Zaki, Heba Diab

**Affiliations:** 1 Department of Medical Genetics, Faculty of Medicine, Umm Al-Qura University, 21955 Mecca, P.O. Box 57543, Kingdom of Saudi Arabia; 2 Department of Molecular Genetics, Faculty of Medicine, Medical Genetics Center, Ain Shams University, Cairo, Egypt; 3 Department of Dermatology, Venereology and Andrology, Faculty of Medicine, Ain Shams University, Cairo, Egypt

## Background

Screening of Yq has become one of the most frequently performed postnatal molecular genetic tests in Egypt. A survey sponsored by WHO estimated the prevalence of infertility among Egyptian couples to be 12% (4.3% for primary infertility and 7.7% for secondary infertility) [1]. The 10-Mb AZF region on the q-arm of the Y chromosome is frequently deleted in men with unexplained spermatogenic failure. Microdeletions are linked to AZF loci in 20-30% of patients with non-obstructive azoospermia and in 3-7% of patients with severe idiopathic oligospermia [2]. AZF microdeletions are associated with varied testicular histology, ranging from Sertoli-Cell-Only (SCO) syndrome to hypospermatogenesis to maturation arrest. We aim to determine the tag sequence-tagged sites (STSs) in the AZF-region of Yq associated with azoospermia and severe oligospermia in infertile Egyptian men.

## Materials and methods

We analyzed buccal cells from 98 infertile Egyptian men with average ages 22-45 years (56 with azoospermia plus 42 with severe oligospermia) using multiplex PCR for six common AZFa, AZFb, and AZFc STS markers.

## Results

Forty-eight (37%) microdeletions with five separate deletions were identified. We found 66.7% of the deletions in the AZFb locus, 20.8% in the AZFa locus, and 12.5% in the AZFc locus. Some common haplotypes (7 of 10) were identified in our sample population. Haplotypes H3 (sY127) and H4 (sY134) were the most common. Separate microdeletions were interestingly localized in infertile Y-chromosome patients.

## Conclusions

We conclude that a minimum of three tags; STSs-sY86, sY127 and sY134 can be used to screen infertile Egyptian men for Yq microdeletions before assisted reproduction is initiated as a treatment.
